# Green biologically synthesized metal nanoparticles: biological applications, optimizations and future prospects

**DOI:** 10.2144/fsoa-2023-0196

**Published:** 2024-05-15

**Authors:** Radwa N Morgan, Khaled M Aboshanab

**Affiliations:** 1 National Centre for Radiation Research & Technology (NCRRT), Drug Radiation Research Department, Egyptian Atomic Energy Authority (EAEA), Cairo, 11787, Egypt; 2 Microbiology & Immunology Department, Faculty of Pharmacy, Ain Shams University, Cairo, 11566, Egypt

**Keywords:** antibacterial, anticancer, green synthesis, metal nanoparticles (MNPs), microbes, plants

## Abstract

In green biological synthesis, metal nanoparticles are produced by plants or microorganisms. Since it is ecologically friendly, economically viable and sustainable, this method is preferable to other traditional ones. For their continuous groundbreaking advancements and myriad physiochemical and biological benefits, nanotechnologies have influenced various aspects of scientific fields. Metal nanoparticles (MNPs) are the field anchor for their outstanding optical, electrical and chemical capabilities that outperform their regular-sized counterparts. This review discusses the most current biosynthesized metal nanoparticles synthesized by various organisms and their biological applications along with the key elements involved in MNP green synthesis. The review is displayed in a manner that will impart assertiveness, help the researchers to open questions, and highlight many points for conducting future research.

## Plain language summary

Metal nanoparticles are small sized particles with diameters ranging from 1 to 100 nm. These particles have favorable characteristics that made them substitute regular sized particles in various industrial fields. They can be prepared chemically or physically or biologically. Biological preparation of metal nanoparticles which is also known as green synthesis involves the use of different microbes and plant species. It's more beneficial than chemical and physical preparative methods, as it's considered cheap and environmentally sustainable. This review will summarize different forms of green synthesis of metal nanoparticles and their application in biomedical field.

## Abstract (plain language summary)

Nanomedicine has solved many drawbacks in therapeutics and diagnositics.

Chemical and physical metal nanoparticles (MNPs) synthesizes are energy consuming and sometimes associated with accumulation of toxic byproducts.

Green biological MNPs synthesis offers an energy saving and cost effective alternative synthesizing procedures.

Green biological MNPs synthesis utilizes plants, bacteria, fungi, viruses and algae as MNPs nanofactories.

Green biological MNPs acquire anticancer and antimicrobial activities due to reactive oxygen species (ROS) generation.

Green biological MNPs attain desirable morphologies that enhance their biological activities.

AuNPs, AgNPs, ZnO-NPs, CuO-NPs, TiO_2_-NPs and IONPs derived from different biological sources acquire significant antimicrobial, anticancer and antioxidant properties.

Biogenic SeNPs are promising therapeutic agents when used alone or incombination with irradiation sources.

Biogenic PtNPs retrieved from fungal biomasses acquire promising physiochemical properties for various therapeutic applications.

Physical factors such as time, pH, temperature, concentration of precursor solution and biological biomasses affect the yield and physiochemical properties of biogenic MNPs.

Optimization of synthesizing technique is essential prior to scaling up production of biogenic MNPs.

Actinomycetes are potiential MNPs nanofactories that need to be investigated.

Statistical optimization techniques and genetic studies may improve optimization techniques and the scaling up of green MNP synthesis.

ADPT, hyperthermia and radiotherapies can be combined with green biogenic MNPs.

Toxological studies for Green MNPs should be properly investigated.

Nanomedicine has revolutionized traditional treatments and diagnostic approaches. It elevated the efficacy of drug delivery, biomarker detection sensitivity, diagnostic imaging and lowered drug-associated side effects [1]. Several nano-based therapies have been already introduced into the market, particularly those targeting cancer and microbial pathogens [2]. It's interesting to note that metal nanoparticles (MNPs) showed advantageous optical, morphological and physiochemical characteristics that opened up new opportunities for nanomedicine, notably in the field of targeted drug delivery systems. They serve as transporters for many medicinal substances, antibodies, nucleic acid fragments etc. Through various forms of bonding, their external surfaces operate as interactive areas that promote conjugation with different forms of bioactive compounds [3,4].

MNPs were created using numerous chemical, physical and biological synthesizing techniques. The chloroauric acid, chloroplatinic acid and silver nitrate are chemical reducing agents that are used in the fabrication of gold, platinum and silver nanoparticles, respectively. The physical synthesis of MNPs incorporates different forms of irradiation (such as gamma rays, UV), combustions (i.e. thermal) and pyrolysis techniques [5]. Although MNP can be produced efficiently and inexpensively through chemical and physical means, current research is focused on creating green, environmentally friendly MNPs by incorporating biological processes [6]. This could be explained by the dangerous byproducts and high energy requirements involved in the chemical synthesis of MNPs. Candidate MNPs for medicinal usage, is constrained by the possibility of contamination from the chemical solvents employed during the fabrication process [7,8]. The chemical solvents residues themselves could be carcinogenic, mutagenic and cytotoxic [9]. Additionally, variations in the physiochemical characteristics and morphologies of chemically and physically manufactured MNPs are linked to various toxicities. Gold nanoparticles (15 nm) were lethal to fibroblasts, epithelial, macrophage and melanoma cell lines [10]. Compared with neutrally charged nanoparticles, the positively charged gold nanoparticles reduced the cell survival of mouse breast cancer 4T1 cells by 50% [11]. This is assigned to the increased electrostatic attraction with negatively charged cell surfaces and enhanced cellular uptake of these particles, which was associated with high levels of oxidative stress and intracellular reactive oxygen species (ROS) [11]. Further, needle and plate shaped nano-sized hydroxyapatite are more hazardous than sphere- and rod-shaped nanoparticles due to their high cell membrane penetrative abilities that compromise cells integrity incurring high levels of cytotoxicity [12]. In similar veins, nano spike titanium dioxide nanoparticles induced potassium efflux and inflammasome activation in macrophages and dendritic cells [13].

Microbial or plant cellular mechanisms that reduce metal ions into their elemental form are used during green biological synthesis of MNPs. In contrast to the intracellular green MNPs synthesis, which involves delivering ions inside the microbial cell where they interact with enzymes to produce nanoparticles, the extracellular MNP synthesis traps the metal ions at external cell surfaces of microorganisms for conversion to its elemental form [14,15]. MNPs created through biological mechanisms are preferable to those created through chemical techniques in several ways [16]. The biological processes use less energy and do not require expensive chemical precursors, making them a better option for the environment. Additionally, biogenic MNPs acquire favorable physiochemical characteristics, greater surface area to volume ratios, regular forms (spherical) and optimal sizes [16,17]. Green MNPs have demonstrated lesser toxicities and adverse effects than chemically induced MNPs. Green silver nanoparticles (AgNPs) elucidated a lower rise in a number of hematological indicators of treated fish when compared with chemically prepared AgNPs [18]. At relatively modest doses (6.25–25 μl), the *Ferula asafetida* AgNPs were fatal to lung and cervical carcinoma cell lines and down-regulated the expression of PIK3CA and KRAS oncogenes, indicating their efficacy, safety and tolerability [19]. When evaluated at low doses (10 mg/Kg), gold nanoparticles (AuNPs) isolated from Egyptian propolis extract exhibited notable antibacterial, anticancer, and anti-mycobacterial effects while evidencing minor toxicities and organ damage within albino rats [20]. The *Cymbopogon citratus* iron oxide nanoparticles' reduced inhibitory effects on the motility, growth and reproduction rates of *Caenorhabditis elegans* worms served as evidence that they were biocompatible and safe for environmental usage [21]. Applications of biological MNPs have included cancer therapy, antimicrobial agents and diagnostics [22,23]. Today, the paradigm change from conventional manufacturing methods to green MNP production is entwined with the concepts of sustainability and energy-saving principles [24].

In this review, different methods of green MNP production through biological green processes will be briefly reviewed before moving on to the optimization techniques. It will also discuss the role of green MNPs as potential anticancer and antimicrobial agents. Future prospects for green MNPs synthesis and applications will be covered in the final section.

## Nanoparticles in biological therapies

Numerous industries, including medicine, have experienced major transformations thanks to the introduction of nanotechnology. It enabled the creation of molecules within the submicroscopic ranges by manipulating atoms and taking advantage of naturally existing quantum phenomena within the nanoscale level. The fabricated nanoparticles have advantageous chemical, physical and biological features that made them outperform their bigger, or ‘bulk,’ counterparts' molecules [25]. It is heavily influenced by developments in cellular biology and molecular genetics, and focuses on enhancing physiological processes at the nanoscale. These nanoscale molecules can easily internalize into the cells, and readily integrate with different cellular elements (e.g. receptors, nucleic acids, antibodies, cellular proteins) [26]. Their nanoscale size also enables their simple diffusion across diverse tissues and organs and facile movement within blood circulation, which is vital for targeted administration of therapeutics and reduces their negative effects [27]. Nanotherapeutics have proven superiority over traditional therapies in various aspects (absorption, toxicity and solubility) and been recently implemented in various medical settings, to enhance effectiveness, safety, sensitivity and personalization [28].

**Figure 1.**
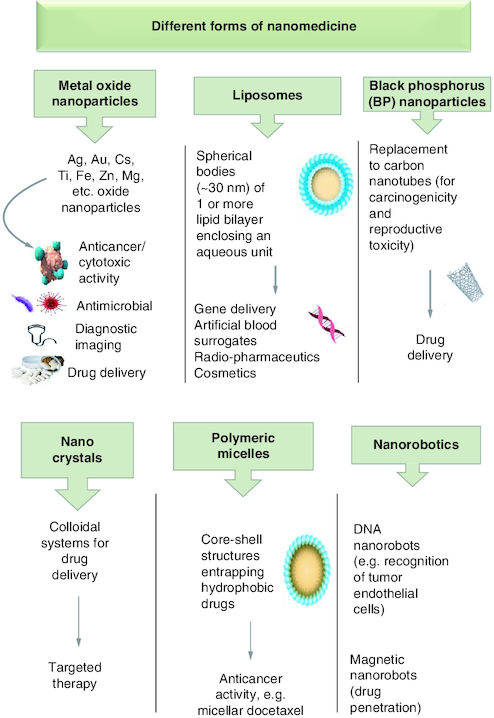
Different forms of nanomedicine and their biological applications.

Nanomedicine has been implemented in three major medicinal applications, therapeutics, diagnostics and theragnostic. Therapeutic nanomedicine uses nanoparticles to treat ailments, while in diagnostic nanomedicine, nanoparticles aids in the early detection of diseases via noninvasive techniques or the evaluation of disease biomarkers. Theranostic nanomedicine is a cutting-edge form of nanotechnology that simultaneously performs therapy and diagnosis [28]. There are various forms of nanoparticles that have been used in nanomedicine (Figure 1). They can be made of inorganic materials such as metal oxide and black phosphorus nanoparticles, and carbon nanotubes, dendrimers, micelles, liposomes, nanocrystals, etc. They had been incorporated in various medicinal applications and proven more efficacious than conventional therapies. For instance, AgNPs of different sizes (1–100 nm) displayed antibacterial properties against pathogenic Gram-positive and -negative bacteria [29]. They also acquire anticancer activity against various cancer lines (e.g. colon cancer cell HCT-116) [30]. Gold nanoparticles (AuNPs) are known for their anticancer activities against various cancer cell lines (e.g. 5637 human bladder carcinoma cells) [31]. Further, liposomal encapsulation of celecoxib reveals a threefold increase in tissue accumulation within BALB/c mice harbouring C26 colon cancer cells, suggesting the high biocompatibility of liposomal nanodrugs [32]. The hazardous carbon nanotubes have been substituted by black phosphorus nanosheets (BPNS) for their high loading capacity and biocompatibility [33].

## Benefits & drawbacks of nanoparticles in nanomedicine

Nanomedicine offers several advantages over conventional treatments. Drugs and therapeutic agents can be delivered to specific disease areas, minimizing the chance of adverse effects and maximizing the therapeutic impact. To address ulcerative colitis, a dual-targeted nanoparticle-in-microparticle delivery system known as Asiatic acid-loaded delivery method (AA/CDM-BT-ALG) was developed to prevent early drug release and degradation in the gastrointestinal environment [34]. Additionally, nanotechnology-based imaging techniques allow earlier and more accurate diagnosis of carcinoma metastasis [35]. Nanochains made of iron oxide NPs coupled with cRGD enhanced the magnetic resonance imaging (MRI) signal and successfully detected micro-metastases of relatively low diameter (<0.5 mm) [36]. Technetium-99m (99mTc) was utilized as a radionuclide label for MNPs (AuNPs) during gamma scintigraphy procedure. Within only 30 min of injecting 99mTc-AuNP radiotracer, gamma scintigraphy identified 4T1 breast carcinoma metastasis in *in vivo* animal models [37].

NPs can improve drug delivery systems, reduces toxicities and cause undesirable side effects. For instance, piperlongumine (PL), an alkaloid that revealed pronounced cytotoxicity against cancer cells, had its water solubility and bioavailability increased by albumin nanoparticles (BSA-NPs) [38]. Nanoparticles can also be used to deliver growth factors and regenerative agents to damaged tissues to help with tissue repair and regeneration. Tantalum nanoparticles (TaNPs) and nanoscale magnesium oxide (MgO) were added to poly caprolactone (PCL)-based periosteum replacement nanofiber to improve its osteogenic and angiogenic capabilities that may heal destroyed periosteum. The PCL/Ta/MgO nanofiber elucidated high cytocompatibility and enhanced osteogenesis and angiogenesis capabilities [39].

Nevertheless, as the use of nanoparticles in medicine is still a subject of research, less is known about their long-term toxicity. For instance, carbon nanotubes are well-known for their extraordinary mechanical and electrical capabilities; however, they resemble asbestos which make them potentially harmful. For their graphitic nature, they are anticipated to be biologically persistent in the body [40]. Coated magnetic nanoparticles including super-paramagnetic iron oxide (SPIONs) may aggregate within the tissues [41]. Studies have also shown that exposure to metal nanoparticles over extended periods damages DNA, which predisposes to cancer and developmental toxicities. They can migrate across the dendrites and axons, inducing oxidative stress and inflammatory responses [42]. Histological examination of mice brain tissues elucidated nanoparticle aggregates, but no obvious toxicity or functional disruptions were reported [43]. Therefore, strict regulatory permissions are necessary prior to usage of MNPs in clinical trails which may delay their industrial usages [44].

## Conventional metal nanoparticles synthesis techniques & the introduction of green synthesis technology

Numerous chemical and physical processes are used for MNPs synthesis, but their biological relevance is restricted due to the usage of toxic compounds as reducing agents for precursor materials. According to Li *et al.*, chemical and physical methodologies are complex and may accumulate toxic byproducts that are harmful to both environment and human health. In order to increase the therapeutic applications of MNPs, environmentally friendly and nontoxic manufacturing procedures are perquisite [22]. The green syntheses of MNPs utilize safe, non-toxic and environmentally friendly reagents. Further, the green biogenic MNPs have advantageous physiochemical natures, higher stability and optimal dimensions [45].

**Figure 2.**
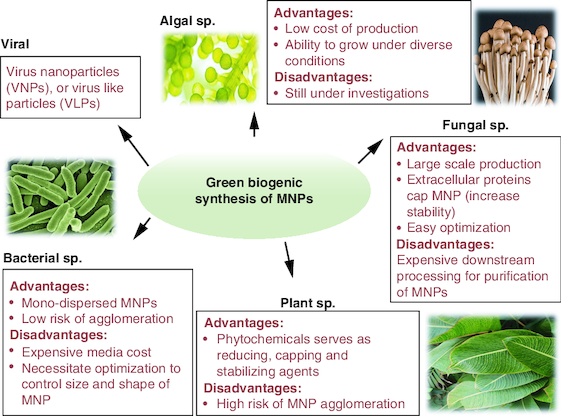
Advantages of green synthesis of metal nanoparticles. MNP: Metal nanoparticle.

Green nano-biotechnology is generally defined as the use of microbes and plants to synthesize nanoparticles via intracellular or extracellular biological processes [46]. It offers alternative pathways for creating stable, eco-friendly metal nanoparticles (green biogenic MNPs) (Figure 2) [47]. The advantages and disadvantages for the commonly used chemical, physical and green approaches MNP synthesizing approaches are summarized in Table 1.

**Table 1.** Common methods for synthesizing green, physical and chemical metal nanoparticles, along with their benefits and drawbacks.

MNP synthesis method	Basic principle	Applications	Advantages	Disadvantages	Ref.
Chemical methods					
Bottom up chemical methods					
Chemical vapor deposition (CVD)	A chemical reaction taking place either on or near a heated substrate which results in solid compound depositions from vapor The deposited substance might be a single crystal, thin film or both	Carbon-encapsulated cobalt (Co@C) NPs (excellent ferromagnetic properties) CdSSe nano-flowers (CdSSe NFs) (Surface-enhanced Raman scattering substrate for antibiotics detections)	• Synthesis of coatings with consistent thickness and low porosity • Allow selective or local deposition of material on patterned substrates.	• Relatively high temperature of gases and substrate to facilitate the deposition (hazardous and energy consuming) • Long cycle time (long heating and cooling periods)	[^48-50^]
Langmuir–Blodgett technique	Generation of supra-molecular assemblies in ultrathin films	Gold and silver nanoparticles (AuNPs and AgNPs)	Creation of well-ordered mono and multi layered molecular structures	• Long deposition times • low reproducibility	[51,52]
Spray Pyrolysis	It involves a heated substrate, an atomizer and metal precursor solution Generation of MNPs thin films due to atomization of precursor solution	High entropy Mn, Zn, Ni, Cu and Fe oxide NPs	• Simple process • Cost effective • Continuous operation with high yield.	• Yield is relatively low • Difficult optimization procedures	[^53-55^]
Sol-gel method	Heating metal alkoxide solution until it reaches gel consistency followed by appropriate drying technique	SiO_2_-NP nano-carriers in in drug/gene target delivery and imaging diagnosis	• High purity • Narrow NPs particle size • Uniform nanostructure distributions	• Cost of the raw chemicals is high • Volume shrinkage and gel cracking have been reported while drying	[56,57]
Solvo-thermal method	Both non-aqueous solvent and precursor substances are heated (>boiling point) within a closed system	ZnO-NPs with ammonia sensing capabilities	High temperature and pressure facilitate the solvation of any substance	• Usage of non-environmentally safe and toxic solvents • Discontinuity of the process due to prolonged reaction times, and uneven heating to obtain desired material.	[58,59]
Co-precipitation method	Simultaneous precipitation of hydroxide form of MNPs from precursor salt in the proper solvent. The oxide MNP is obtained by drying. Multistage process involves nucleation, growth, coarsening, agglomeration and ripening.	Zinc ferrite nanoparticles, ZFO NPs (prominent cytotoxic activities)	• Most practical, affordable method for synthesis of MNPs • Mono-dispersed NP.	Continuous washing, drying is perquisite to obtain metal NPs	[60,61]
Micro-emulsion method	When two non-nucleated micelles collide, the entire solute is transferred to one micelle	Magnetic Fe_3_O4@polydopamine core-shell NPs (Fe_3_O_4_@PDA) for targeted chemotherapy	• High efficacy • Affordability • High purity • High efficiency • Easy optimization	Small linear range of nanoparticles near micromolar doses, necessitating sample pre-dilution	[62,63]

Top-down chemical methods					
Metal Assisted Chemical Etching (MACE)	Semiconductors (silicon) are chemically etched with the help of a metal catalyst	Gold nanoparticles	High surface contrast	• High chemical disposal costs • Poor process control (temperature sensitivity) • Weak anisotropy and poor particle size control	[64,65]

Physical methods					
Top-down physical methods of synthesis					
Laser Ablation (LASiS)	Laser irradiation of liquid submerged target	Biocompatible AgNPs	• Considered green technology (low costs) • A powerful means of cleaning surface without the use of any chemicals.	• Hazardous emissions due to laser vaporization and substance combustion (necessitate reliable exhaust system) • Lasers beams may inflect serious fire and health risks.	[66,67]
Sputtering	Bombardment of target material with intense gas or gaseous plasma ions, resulting in formation of small particles of the target material emission.	Pt/Ag solid solution alloy NPs (<3 nm)	• Existence of reactive plasma with high oxidizing potential. • Relatively low cost	The bombardment's target area is relatively low, and the average deposition rate is poor.	[68,69]
Mechanical milling	Factors such as milling method (ball or attrition miller), applied power, milling medium (such as tungsten carbide balls), control solution (such as toluene), mill speed and time all affect the properties of NPs	Al-5Al_2_O_3_ nanocomposites	High capacity to manufacture a wide range of NPs	• Noisy procedure • Relatively slow and exhausting • Soft fibrous material cannot be milled by ball mill.	[70,71]

Green synthesis					
Plant	Different parts of plants, leaves, roots, seeds utilized for NPs synthesis	Au, Ag, TiO_2_, ZnO, Pt/Pd NPs (anticancer, antimicrobial, antibacterial, etc.)	• Low cost, less pollution, eco-friendly • Plants phytochemicals that may serve as organic stabilizing and/or reducing agents for MNPs • Less toxic MNPs with high biological potential.	• May not be suitable for large scale productions • Poly-dispersed MNPs of varying particle size • Further investigations are required to assess activities and properties of MNPs	[72]
Bacteria	Various bacterial sp. (*Bacillus subtilis, Klebsiella pneumonia, Escherichia coli, Lactobacillus* sp.*, Brevibacterium frigoritolerans*)		• Environmentally benign and nontoxic synthesizing method. • Simple to manipulate and control the physical and chemical. • Generate metal nanoparticles that are highly stable and have less agglomeration propensity (attributed to capping of NPs by natural microbial proteins) • Mono-dispersed MNPs and any irregular sizes may be adjusted by strict control of physical parameters	• Cost of media and bacterial isolation and identification might be expensive • Long production time • Synthesis is highly dependent on physical parameters (pH, Temp, etc.) which necessitate optimization	[73,74]
Fungi	Various fungal sp. *(Aspergillus* sp. *Colleotrichum* sp. *Rhizopus* sp. *Fusarium* sp. *Guignardia sp.*)		• Excellent metal tolerance • Secretion of high conc. of extracellular proteins that cap and stabilize MNPs • Fungal cultures are more beneficial over bacterial system (strong biomass production) • Fungal mycelial mass is agitation and pressure resistant • Large-scale syntheses of MNPs and easy manipulation of metabolism to control production of MNP	Multistage downstream process for the retrieval of the NPs that might be expensive	[75]

Al: Aluminum; Au: Gold; Ag: Silver; Cu: Copper; MNP: Metal nanoparticle; NP: Nanoparticle; TiO_2_: Titanium dioxide; ZnO: Zinc oxide.

## Green metal nanoparticles produced by plants species & their biological applications

Plants are regarded low-maintenance and cost-effective MNPs nanofactories [76]. Various plant components have been used for MNPs production since their extracts are rich with phytochemicals that may act as reducing and stabilizing agents during MNPs production [8,76]. To synthesize nanoparticles through plants, the selected plant portion is thoroughly washed and boiled in distilled water. The precursor solution for the required MNP synthesis is mixed with the plant extract after consecutive steps of squeezing and filtration. Following the addition of MNP precursor solution, the color of the plant extract begins to change which indicates the formation of MNPs [77].

Phytochemicals in plants biomasses act as both reducing and capping agents for metal nanoparticles [78]. Functional moieties found in phytochemicals such as OH, carboxyl and ketones, act as bio-reducers for phyto-MNPs whereas plant proteins and amino acids act as capping agents. Terpenoids, alkaloids, tannins, phenolic acids and flavonoids are powerful reducing and stabilizing agents. Protein and amino acids capped MNPs elucidate favourable physiochemical properties and stability profiles and less prone to aggregation [79].

The isoprene based terpenoids and flavonoids are capable of reducing both silver and gold salt solutions to nanoforms. The eugenol-rich clove extract harnessing hydroxyl groups bound to benzene rings together with potent electron-withdrawing groups at the ortho and para positions, methoxy and allyl groups, successfully reduced AuCl_4_ and AgNO_3_ to Ag and Au-NPs. The strong electron withdrawing groups, methoxy and allyl, surrounding the hydroxyl group enhanced the ability of eugenol to release protons making it an extremely potent reducing agent. Further, the release of proton by eugenol is stabilized by a resonating anionic form. The electrons are up-taken by both AuCl_4_ and AgNO_3_ generating the reduced forms Au^0^ and Ag^0^ [80]. On the other hands, flavonoids rich *Ocimum basilicum* and *Mangifera indica* leaf extract emitted protons in their tautomeric state (transformation from enol to keto state) that reduced silver salts and generated AgNPs [81]. Gold nanoparticles were retrieved from extracts of *Magnolia kobus* and *Diopyros kaki* following the reduction of aqueous HAuCl_4_ solution by the ketones, alcohols, amines, aldehydes and carboxylic acids [82]. The hydroxyl group of quercetin in broccoli extract reduced the calcium ion to Ca(OH)_2_, which is then dried and calcined to CaO nanoparticles [83]. Dihydromyricetin is also thought to be involved in the production of AuNPs through the oxidation of hydroxyl to carbonyl groups [84]. Aqueous ginger/cardamom extracts are now recognized promising phyto-MNPs generators as they are opulent with various capping, reducing/stabilizing and chelating agents (e.g. ginerol, luteolin, quercetin, rutin, etc.). Using iron nitrate, cobalt nitrate and aqueous extracts of ginger and cardamom, crystalline cobalt ferrite of an average size 100 nm can be successfully synthesized [85].

Alteration in plant extract content during phyto-MNPs production impact the morphological and physiochemical characteristics of MNPs. Higher plant extract concentrations yield spherical MNPs of uniform distribution, while lower concentrations yield bigger, poly-dispersed phyto-MNPs. In contrast to hexagonally formed AuNPs of >100 nm at low extract concentrations, Lin *et al.* reported the appearance of spherical AuNPs with a diameter <15 nm among high *Morinda lucida* leaf extract concentrations. This is assigned to relatively high concentration of phytochemicals in higher plant extract concentrations necessary for MNPs capping and prevention of aggregation [86]. The chemical composition of the plant varies according to the plant part, wherefore; the section chosen for phytosynthesis will also affect MNPs properties. Appapalam and Panchamoorthy denoted the presence of regular sized AgNPs (<15 nm) in the *Aerva lanata* leave extract while larger AgNPs were prevalent among *A. lanata* flower extract [87]. The *A. lanata* extracts are opulent with flavonoids (rutin, quercetin and kaempferol), polyphenols (gallic acid and ellagic acid) and saponins that were involved in the reduction of AgNO_3_ [87]. Additionally, plant extract storage conditions also influences size and dispersion of phyto-MNPs. Storage conditions affect the phytochemicals stabilities regulating the bio-reduction and capping of biogenic MNPs which in turn affects the bio-reduction process. Elevating the storage temperatures raises the plant extract aging time and destabilizes the phytochemicals. This is associated with large poly-dispersed phyto-MNPs [79]. Table 2 summarizes the different types of MNPs synthesized by various plant extracts.

**Table 2.** Metal nanoparticles synthesized by different plant extracts.

MNP	Producing organism	MNP characteristics	Requirement for production	Ref.
Silver nanoparticles (AgNPs)	• *Saccharum ofcinarum* *• Helianthus annus* *• Cinamomum camphora* *• Oryza sativa* *• Aloe vera* *• Capsicum annuum* *sativa* *• Magnolia Kobus* *• Hibiscus rosa sinensis*	• Unique size-related physical and chemical characteristics. • Favorable optical characteristics • Favorable electrical and thermal conductivity • Favorable biological and catalytic activities	Plant extract co-incubated with silver metal ion solution (AgNO_3_)	[88,89]
Gold nanoparticles (AuNPs)	• Geranium leaf extract • *Aloe vera* leaf • *Magnolia kobus* leaf extracts • *Cofea Arabica* • *Croton sparsiforus* leaves extract	• AuNPs with excellent optical properties • Adjustable AuNP sizes by changing the gold precursor and plant extract concentrations.	Precursor salt solution (AuCl_3_) mixed with plant extracts	[77,^90-92^]
Zinc oxide nanoparticles (ZnO-NPs)	• Flower and leaf of *Vitex negundo* • *Hibiscus subdariffa* leaf extract	• ZnO-NPs with high semiconducting properties • High enzymatic activity, wound healing, anti-inflammatory, and ultraviolet filtering properties. • ZnO-NPs with enhanced photocatalytic activity.	Mixing plant extract with a 0.5 mM of either hydrated zinc sulphate, zinc oxide, and zinc nitrate.	[93,94]
Pd/Pt nanoparticles	• *Catharanthus roseus*	• Pd and Pt NPs with high surface energy and high surface area to volume ratio, facilitating their action as catalysts • Low genotoxicity • High yield of Pd/Pt-NPs	Aqueous solution of palladium acetate mixed with methanolic extract of plant biomass.	[^95-97^]

MNP: Metal nanoparticle.

## Plant derived metal nanoparticles as anticancer agents

Numerous MNPs fabricated by plants species elucidated anticancer potential through the activation of apoptotic pathways and regression of angiogenesis. The AgNPs incur double or single DNA strand breaks and induce apoptosis. They also halt mitochondrial respiration blocking ATP synthesis and disrupt the activity of the RNA topoisomerase enzyme hindering gene transcription [98]. Further, *Houttuynia cordata* CuONPs stimulated HeLa cells apoptosis by acting upon the PI3K/Akt signaling pathways [99]. It is also thought that AuNPs are capable of binding to VEGF hindering its integration with VEGFR receptor which reduces angiogenesis. The VEGF/VEGFR-stimulated angiogenesis activates three downstream pathways, the MAPK, JNK/c-Jun and AKT signaling pathways. Downregulation of either VEGFR or VEGF disturbs these downstream pathways, which results in anti-angiogenesis effect [100,101].

MNPs results in the production of reactive oxygen species (ROS), which induces oxidative stress, dsDNA, protein denaturation and p53-mediated intrinsic apoptosis [102]. Biogenic AgNPs upregulate the expression caspases 8, 9 and 3 apoptosis factors in lung cancer cell lines [103]. Subcellular morphological alterations, such as cell shrinkage, clumping, dissociation, rounding and the generation of apoptotic bodies, are also linked to the use of MNPs against cancer cells [104]. MNPs also induce membrane leakage as noted by extensive leakage of lactate dehydrogenase (LDH) from biogenic AgNPs treated cells [105]. Moreover, biogenic AgNPs can change the tumor's metastatic properties and diminish the microvilli growth [106].

Crude peel extract of *Garcinia mangostana* (GM) was used to fabricate gold–silver core shell nanoparticles (Au–AgNPs) for targeted delivery of protocatechuic acid (PCA). The dried extracts of GM were mixed with precursor salts, tetrachloroauric (III) acid (HAuCl_4_) and silver nitrate (AgNO_3_) to produce Au–AgNPs. The GM extract Au-AgNPs were spherical with only few irregularities. The IC_50_ values for GM Au-NPs and Au-AgNPs against colon carcinoma HCT116 cells were 82.99 and 24.36 g/ml, respectively. Loading the core shell Au-AgNPs with PCA was associated with eminent reduction in IC_50_ value. Against the HCT116 colon cancer cell line, 15.6 g/ml Au–Ag-PCA-NPs demonstrated effective suppression (70%) while remaining non-lethal to normal colon CCD112 cells denoting their selectivity and superiority [107]. In similar manners, Fe_3_O_4_ nanoparticles (Fe_3_O_4_-NPs) were generated using the fruit peel extract of *Garcinia mangostana*. The produced Fe_3_O_4_-NPs were lethal to colon cancer HCT116 cells and benign to normal colon CCD112 cells indicating that *G. mangostana* fruit peel extracts can be utilised as an inexpensive stabilising and capping agent for the manufacture of Fe_3_O_4_-NPs [108].

AuNPs and AgNPs can also be synthesized by GM pericarp waste extract. In this green synthesis process, the hydroxyl groups prevalent among GM extract acted as reducing agents for the reduction of gold or silver salts. The GM extracts produced spherical AuNPs (15.37 nm) and asymmetric AgNPs (13–31 nm). Interestingly, only GM-AgNPs were cytotoxic to lung carcinoma A549 and mouse fibroblast NIH3T3 cells lines. GM-AuNPs, on the other hand, may be used as drug-delivery carriers since they are benign to cultivated cell lines [109].

Biofabricated CuO-NPs with anticancer activity were retrieved after utilizing the fruit extract of *Prunus nepalensis*. The CuO-NPs were crystalline in nature (∼35–50 nm) and were able to upregulate *p53*, *Bax*, *caspase-3* and *caspase-9* genes and down regulate *Ras* and *Myc* oncogenes in *in vitro* cultured MCF-7 cells [110]. In similar manners, bio-fabricated CuO-NPs by *Prunus mahaleb Leaf* extracts were spherical (20–30 nm) with antioxidant, anticancer, anti-mutagenicity and antimicrobial potential against Gram-positive bacteria [111]. *Pimenta dioica* biofabricated CuO-NPs were spherical (20–50 nm) with prominent antibacterial activity when tested on *S. aureus, S. mutans, P. aeroginosa* and *E. coli.* They also exhibited lethality to *in vitro* cultured human colorectal cancer cells and anti-diabetic potentiality through *in vitro* alpha amylase inhibitory assay [112].

The *Raphanus sativus var. Longipinnatus* biofabricated ZnO-NP exerted anticancer activity against A549 cell lines [113]. The reducing agents found in *C. crista* seed extract, such as alcohol, phenol and alkyl halides, enabled the biofabrication of crystalline ZnO-NPs conffering significant antibacterial activities against 14 microorganisms, cytotoxicity against HeLa, MCF-7 and regular fibroblast cell lines [114]. Uniform spherical green AgNPs with antioxidant and anticancer capabilities against the alveolar carcinoma cells (A549 cells) can be obtained from *Leucus aspera* extract [115].

Yuan *et al.* have retrieved nickel nanoparticles (Ni-NPs) from the extracts of *Alhagi maurorum* (camelthorn) with anticancer activity against various malignant ovarian carcinoma cells while being benign to normal HUVEC cells [116]. In similar manners, Huang *et al.* biofabricated spherical Ni-NPs with potent anti-ovarian caner activity from the aqueous extract of *Fumaria officinalis* [117].

## Plant derived antimicrobial metal nanoparticles

One of the most urgent challenges of our decade is the surge of antibiotic resistance. *Cassia fistula* and *Melia azadarach* ZnO-NPs (spherical, 3–68 nm) elucidate antibacterial potential against *E. coli* and *S. aureus*. Standard antibiotics and biogenic ZnO-NPs were evaluated, and the results revealed that the later had a stronger antibacterial vigor [118]. In similar manners, *Cassia alata* spherical ZnO-NPs (∼60 nm) elucidated antibacterial activity against *E. coli* with an IC_50_ value of 20 μg/ml [119]. The dried leaves extracts of *C. auriculata* generate spherical biogenic ZnO-NPs (20–30 nm) that repressed *B. subtilis, K. pneumoniae, P. aeruginosa* and *Proteus mirabilis* [120]. Sustainable ZnO-NPs purified from the ethanol extract of *Maesa indica Roxb. Sweet (ME)* aerial parts exhibit potent antiviral activity against coronavirus 229E [121].

Silver has long been recognized for its ability to combat numerous bacterial types. A range of pathogenic bacteria were successfully suppressed by *Carissa carandas* AgNPs [122]. Stevia leaf and Saudi Arabian desert plant *Sisymbrium irio* AgNPs suppressed the growth of MDR Gram-negative pathogens [123]. The *Chenopodium formosanum-*mediated AuNPs fabrication yielded AuNPs (6–8 nm) with bactericidal activity against *E. coli* and *Staphylococcus aureus* [124]. *Listeria monocytogenes* and *Serratia marcescens* were likewise sensitive to AuNPs purified from *Trachyspermum ammi* seed extract [125]. *Ginkgo biloba* AgNPs exerted promising anti-HCov-229E and mild anti-MERS-CoV [126].

The protozoan vector-borne disease, leishmaniasis, affects about 350 million people. Leishmaniasis chemotherapeutics regimens are associated with unfavorable side effects. Biogenic AuNPs retrieved from the aqueous extract of *Rhazya stricta decne* acquires anti-leishmanial activity against *Leishmania tropica* (HTD7) amastigotes [127].

Further, all types of *in vitro* cultured ginseng cell lines reduced silver nitrate into spherical AgNPs. *Fusarium graminearum, F. avenaceum, F. poae* and *F. sporotrichioides* are wheat affecting fungal pathogens that were successfully eradicated with AgNPs purified from *Panax ginseng's* hairy root extracts [128]. In similar veins, the pathogenic bacteria *Agrobacterium rhizogenes, Bacillus subtilis, A. tumefaciens* and *E. coli* are repressed by *Aristolochia manshuriensis* AgNPs [129]. *S. aureus* and *K. pneumoniae* are susceptible to *Rhazya stricta* AgNPs extract [130].

## Plant derived antioxidant metal nanoparticles

The extreme oxidative stress can incur oxidative damage to a variety of cell macromolecules, including DNA, proteins and membrane lipids, resulting in degenerative illnesses and ageing [131]. The *Harrisonia abyssinica* AgNPs [132], *Canthium inerme* AgNPs and AuNPs [133], Stevia leaf extract NiO-NPs [134], acquire antioxidant potential.

## Green metal nanoparticles produced by bacterial species their biological applications

Microbial cells can be MNPs bio-factories. They are more frequently utilized for MNPs production for their environmental abundance and exceptional adaptability. Physical and chemical bacterial growth conditions can be easily altered to achieve utmost MNPs production performances [74,135]. Different bacterial species can be utilized for extracellular and intracellular manufacture of MNPs [74,135].

The mechanism of MNPs synthesis often varies among microorganisms, but in general, the process starts with the microbes entrapping metal ions on its external surface or within, followed by bioreduction to MNPs. Typically, entrapment is mediated by electrostatic attraction forces. The bioreduction process is initiated by different intracellular enzymes (e.g. NADH-dependent reductase enzyme) [136]. The extracellular (EC) production of MNPs is the commonly utilized pathway. In EC biofabrication, microbial cell filtrates containing reducing and stabilizing agents are co-incubated with aqueous metallic salt precursor solutions. When metal ion reduction occurs, a visible color change takes place [137]. In case, intracellular technique (IC) is utilized for biogenic MNPs production, the bacterial biomass itself is co-cultivated with the metal precursor salt solution where ion transport ports mediate the metal reduction to MNPs [138]. Bio-fabricated MNPs can be microscopically identified within the periplasmic space, cell wall and cytoplasm of bacterial cells. Intracellular production of AuNPs was observed among *Thermomonospora* sp. and *Rhodococcus* sp. [139].

Furthermore, bacterial supernatants or cultures are capable of capping nanoparticles which halts MNPs aggregation and agglomeration. Capping reduces the MNPs surface energies and maintains their size <100 nm [140]. It's also noted that Gram-positive bacteria have higher silver ion reduction tendencies than the gram-negative bacteria [140]. The Gram-positive *Lactobacillus* sp. are fast and effective AgNPs generators [141]. Co-cultivation of the alkalified *Lactobacillus* sp., *Pediococcus pentosaceus, Enterococcus faecium* and *Lactococcus garvieae* biomass with diamine silver complex generates AgNPs [140]. Table 3 enumerates the features and prerequisite manufacturing methods for the most common bacterially produced MNPs.

**Table 3.** Characteristics and fabrication techniques for the most commonly synthesized bacterial metal nanoparticles.

MNPs	Secreting organisms	Size and shape	Advantageous properties	Fabrication technique	Ref.
Extracellular Ag-NPs	Metal resistant *Bacillus* sp. strain CS 11	Spherical shaped 42–92 nm	Highly stable Extracellular production within 24 h at room temperature	Co-incubation of bacterial cell filtrate with different concentrations of filter-sterilized AgNO_3_ (nitrate reductase aid in MNPs reduction)	[142]
	Thermophilic *Bacillus* sp. AZ1	Spherical shaped 7–13 nm	Small sized Ag-NPs are associated with higher biological activities		[143]
	Pathogenic *Enterococcus aerogenes*	Spherical shaped 47–105 nm	Synergistic effects when combined with antibacterial agents		[144]
	*Bacillus subtilis Escherichia coli*	Spherical shaped 123–323 nm	Stable for long periods		[145]
	Soil *Cuprividus* sp.	Spherical shaped 10–50 nm	Anti-biofilm activity		[146]
	*Bacillus mojavensis* BTCB15	Spherical shaped 105 nm	Antibacterial activity against MDRs	Introduction of Tween 20, and metal ion K_2_SO_4_, for Ag-NP size augmentation with 104% size reduction to 2.3 nm	[147]
Intracellular Ag-NPs	*Lactobacillus casei*	Spherical 25–50 nm or Aggregates 100 nm	–	Co-cultivation of bacterial biomass with electron donor (glucose, 56 mmol L^-1^), and inducer (AgNO_3_, 0.1 mmol L^-1^).	[148]
Extracellular Au-NPs	Cyanobacterium *Leptolyngbya* JSC-1 sp.	Spherical shaped 100–200 nm	Photo-catalytic activity with methylene blue and antibacterial vigor	Co-incubation of bacterial cell filtrate with hydrogen tetrachloroaurate solution (HAuCl_4_)	[149]
	Marine bacterium *Lysinibacillus odysseyi* PBCW2	Spherical shaped 15–35 nm	Relatively stable (∼6 months) Antioxidant Antibacterial Dye-degrading properties (BTB dye)		[150]
	*Nocardiopsis* sp. *MBRC-48*	Spherical shape 11.57 ± 1.24 nm	Antioxidant Cytotoxic/anticancer activity Antimicrobial activity Synergism with other antimicrobial agents	Co-incubation of bacterial filtrate with HAuCl_4_·3H2O	[151]
Intracellular AuNPs	*Enterococcus* sp. RMAA.	Spherical shape	Stable Pro-apoptotic agent/anticancer activity	Co-cultivation of bacterial biomass with AuCl_3_	[152]
Extracellular TiO_2_-NPs	*Bacillus subtilis*	Spherical (66–77 nm) Oval Aggregate (30–40 nm)	Anatase crystalline structure Stable Antibacterial activity	Co-incubation of bacterial cell filtrate with TiO(OH)_2_ solution	[153]
	*lactobacillus* sp.	Spherical aggregates of 40–60 nm	Eco-friendly, low cost Ti_2_O nanoparticles	Co-incubation of cell filtrate with titanium dioxide	[154]
	*Streptomyces* sp.	Spherical 30 to 70 nm	Anti-biofilm activity Antibacterial activity	Co-incubation of cell filtrate with TiO(OH)_2_	[35]
Extracellular ZnO-NPs	Haloalkaliphilic bacterial strain (*Alkalibacillus* sp. W7)	Quasi-spherical shape 1–30 nm	Antibacterial activity against Gram-negative and Gram-positive Antibiofilm activity Photocatalytic degradation for methylene blue and methyl orange dye	Co-incubation of bacterial cell filtrate with precursor solution ZnSO_4_.7H_2_O and zinc sulphate solution	[155]
	*Acinetobacter schindleri*	Polydispersed and spherical in shape	Antimicrobial activity against Gram-positive and Gram-negative bacteria	Co-incubation of cells filtrate with zinc nitrate	[156]
	*Rhodococcus pyridinivorans* NT2	Hexagonal phase, roughly spherical 100–120 nm	Moderately stable Antibacterial activity against aerobic Gram-positive *Staphylococcus epidermidis* NCIM 249 Cytotoxic to colon carcinoma cells	Co-incubation of cell filtrate with ZnSO_4_.H_2_O	[157]
Extracellular CuO-NPs	*Proteus mirabilis* 10B	Rod, needle and wire shaped 17–37.5 nm width 112–615 nm length	Antimicrobial activity against Gram-positive and -negative bacteria Antibiofilm activity	Co-incubation of bacterial cell filtrate with the Cu ion precursor Cu(NO_3_)_2_	[158]
	*Stenotrophomonas* sp. BS95	Roughly spherical particles 35–43 nm	Antibacterial activity Anticancer potential Antioxidant activity	Culture supernatant was co-incubated with copper sulfate pentahydrate	[159]
Extracellular MgO-NPs	Endobacterium *Burkholderia rinojensis*	Roughly spherical granular 26.7 nm	Significant antifungal activity and antibiofilm activity against *Fusarium oxysporum* f. sp. *lycopersici.*	Co-incubation of bacterial cell filtrate with magnesium nitrate (Mg (NO_3_)_2_)	[160]
Extracellular Fe_2_O_3_-NPs	*Bacillus cereus* strain HMH1	Spherical 29.3 nm	Highly Stable magnetic iron oxide NPs Anticancer potiential against breast carcinoma MCF-7 and 3T3 cells	Filtered bacterial supernatant was mixed with FeCl_3_·6H_2_O (5 min) followed by FeCl_2_·4H_2_O (after 30 min)	[161]

## Bacterial derived antimicrobial metal nanoparticles

**Figure 3.**
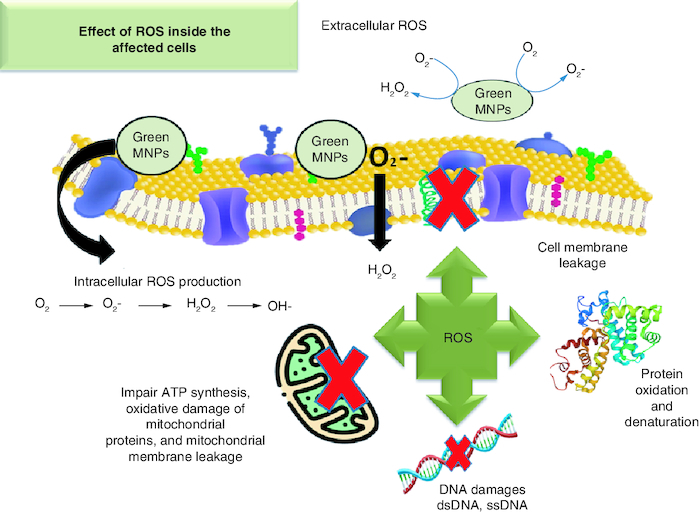
Production of reactive oxygen species by metal nanoparticles inside the affected cells. MNP: Metal nanoparticle; ROS: Reactive oxygen species.

MNP interacts with a variety of microbial targets. Generation of ROS can induce DNA breaks, disrupt proteins translation and harm other essential components (Figure 3). In comparison to traditional antibiotics, nano-antimicrobial agents are both affordable and safe in infectious diseases treatments [74]. MNPs antibacterial activity is heavily influenced by their size and structure. Particles ranging from 1–10 nm exert stronger bactericidal activity than their larger counterparts [162]. Different MNPs with effective antibacterial capabilities were synthesized these included the gold (Au), silver (Ag), titanium (Ti), zinc and magnesium (Mg). Additionally, antibiotic-loaded MNPs enhance the pharmacokinetics of the medicine [163].

## Bacterial derived antimicrobial silver nanoparticles

Bacterial cell wall may be damaged by direct contact with AgNPs, resulting in cellular leakage and cell death. AgNPs are more hazardous to bacteria when they are <10 nm [164]. Pits arise when AgNPs (12 nm) build up on the cell wall of *E. coli*, according to earlier research by Sondi and Salopek-Sondi. These pits lower the *E. coli* outer membrane stability contributing to cellular leakage and LPS and membrane proteins escape [165]. Since Gram-positive and Gram-negative bacteria have different cell wall architectures, Gram-negative bacteria are typically more vulnerable to Ag^+^ invasion. This might be attributed to the fact that gram-positive bacteria have thick cell walls with several peptidoglycan layers that operate as a barrier to the entry of Ag^+^ ions [166,167]. The AgNPs shape and geometry also influences their bactericidal effect. When compared with spherical and rod-shaped AgNP, triangular silver nanoplates exhibit the best biocidal activity [167].

The culture filtrates of endophytic bacterium *Bacillus siamensis* synthesize AgNPs from aqueous silver nitrate (AgNO_3_) when incubated at 30 °C for 24 h. The resultant AgNPs were spherical (25–50 nm) with strong bactericidal effect at 20 μg/ml against the plant pathogens *Xanthomonas oryzae* and *Acidovorax oryzae* [168]. Silver ion tolerant *Bacillus ROM6* synthesizes AgNPs when their bacterial supernatant was co-incubated with AgNO_3_ at 30 °C for 48 h. The resultant AgNPs are mono-dispersed and spherical (<25 nm). These nanoparticles are bactericidal to *S. aureus, E. coli, P. aeruginosa* and *A. baumannii* with minimum inhibitory concentration (MIC) ranging from 1.4 to 5.6 μg/ml. They have also exhibited good stability (∼180 days) [169]. Co-incubation of *K. pneumoniae* supernatants with AgNO_3_ at 37 °C for 24 h generates spherical AgNPs with bactericidal activity [170]. In similar manners, spherical mono-dispersed AgNPs (13.8 nm) were retrieved from the culture extracts of *Lactobacillus* sp. strain LCM5. The retrieved AgNPs are bactericidal to Gram-negative bacteria *Chromobacterium violaceum* with an inhibition zone 18 ± 0.69 mm diameter [171].

## Bacterial derived antimicrobial gold nanoparticles

According to Schrofel *et al.*, AuNPs prevent the binding of tRNA to the ribosomal subunit [172]. They also block unwinding of DNA and mRNA transcription [173]. The bimetallic Ag-Au-NPs purified from *Shewanella oneidensis* co-cultivated with HAuCl_4_ and AgNO_3_ at 37°C are spherical (20 nm) and bactericidal to pathogenic strains*, E. coli, P. aeruginosa, E. faecalis* and *S. aureus*. The lowest recorded MIC is 30 μM and at 10 μM, Ag-Au-NPs elucidate anti-biofilm activity [174].

AuNPs can be retrieved from cultural extracts of hyperthermophilic *Caldicellulosiruptor changbaiensis* co-incubated with HAuCl_4_.3H_2_O for 12 h. The AuNPs have a special quality that set them apart from chemically synthesized AuNPs where the smallest AuNPs attained the highest peroxidase activity over a wide pH range. C. *changbaiensis* AuNPs have impressive antibacterial capabilities and excellent anti-bioflim activities *in vitro* and *in vivo* assays [175]. In similar manners, extracellular spherical AuNPs (20.93 nm) with prominent antioxidant activity are retrieved from cell free extracts of the marine bacterium *Paracoccus haeundaensis* BC74171 [176].

## Bacterial derived antibacterial ZnO, & Fe_2_O_3_ nanoparticles

The zinc-tolerant *Lactobacillus plantarum* TA4 cell-biomass (CB) and supernatant (CFS) are potential nanofactories for ZnO-NPs generation. With particles sizes of 291.1 and 191.8 nm, the biogenic ZnO-NPs have flower-like pattern for ZnO-NPs-CFS and irregular shape for ZnO NPs-CB. They are regarded biocompatible on Vero cell line at low doses and antibacterial to pathogenic bacteria [177]. Using an extracellular approach, spherical iron oxide nanoparticles (IONPs) are created by *Proteus vulgaris ATCC-29905*. Interestingly, *P. vulgaris* IONPs are bactericidal to methicillin-resistant *S. aureus*. The IOPNs have also halted the migration of HT-29 cancer cell in scratch assays and were cytotoxic to glioblastoma cell lines [178].

Secondary metabolites of *Bacillus cereus* HMH1 are also used to extracellularly synthesize spherical IONPs (29.3 nm) [161]. In similar manners, *Staphylococcus warneri* mediate IONP capping by extracellular polysaccharide. The polysaccharide capped IOPNs elucidates high biocompatibility [179]. Spherical IONPs (15 nm) are also produced by *Lactobacillus casei* [180]. *Streptomyces* sp. (SRT12) was employed by Rajeswaran *et al.* to create quasi-spherical IONPs (65.0–86.7 nm) that exhibit strong antibacterial and antioxidant activities [181].

## Bacterial-derived anticancer metal nanoparticles

Gold nanoparticles are biosynthesized by marine bacteria *Vibrio alginolyticus*. These AuNPs acquire strong inhibitory effects on colon cancer cell proliferation with an IC_50_ score of 15 μg/ml. The AuNPs induce apoptotic mediated cellular death and exerts free radical scavenging activity that denotes antioxidant potential [182].

## Green metal nanoparticles produced by fungal species & their biological applications

For their high metal resistance and ability to bio-accumulate metals intracellularly, Fungi are utilized as reducing and stabilizing agents for the production of MNPs. Fungus may readily grow in large industrial scale and generate MNPs with precise sizes and morphologies. Fungal-mediated MNPs production can either be intracellular or extracellular. The metal precursor is added to the mycelial culture biomass in the case of intracellular synthesis. Post synthesis, the nanoparticles must be extracted using chemical processing, centrifugation and filtration to disrupt the biomass and liberate MNPs. On contrast, extracellular fungal biosynthesis of MNPs necessitates the addition of metal precursor to the aqueous mycelial cultures filtrate that solely contains the fungal biomolecules and secondary metabolites. Extracellular fungal biosynthesis of MNPs is the most advantageous techniques since no special processes are needed to purify MNPs from the cells [75].

The enzymes present within the fungal cell extract can reduce metal ions precursors. Nicotinamide adenine dinucleotide (NADH) and NADH-dependent nitrate reductase are involved in the reduction of Ag^+^ ions into AgNPs [183]. By employing *Fusarium oxysporum* to create AgNPs, Durán *et al.* hypothesized that the nitrate reductase enzyme and anthraquinones are responsible for the reduction of silver ions [184]. Further, quinones are also involved in the creation of fungal nanoparticles [185].

According to Hietzschold *et al.*, NADPH alone mediates fungal-mediated nanoparticle formation, which negates the need for optimal nitrate reductase enzyme conditions. This will allow the synthesis of nanoparticles through a variety of species that don't produce reductase enzyme [186]. It's also crucial to note that extracellular metabolites served as stabilizing and capping agents during MNPs production [187]. Table 4 summarizes the characteristics and fabrications techniques for the most commonly fungal synthesized MNPs.

**Table 4.** Characteristics and fabrications techniques for the most commonly fungal synthesized metal nanoparticles.

MNPs	Secreting organisms	Size and shape	Advantageous properties	Fabrication technique	Ref.
AgNPs	Endophytic fungi *Fusarium semitectum*	–	Cytotoxic agent	Filtered fungal extract was mixed with AgNO_3_ at 29 °C for 24 h	[188]
			Antibacterial agent and antibiofilm activity against *Porphyromonas gingivalis, Bacillus pumilus*, and *Enterococcus faecalis*.		[189]
	*Chaetomium thermophilum*	Spherical 8.93 nm.	Safer and less toxic than chemically synthesized AgNPs	Cell free extract was mixed with AgNO_3_ heated at 90°C for 1 h.	[190]
	Fungal extract of *Trametes trogii*	Clustered	Alkaline pH is conducive to the synthesis of AgNPs by fungal species ligninolytic enzymes might be involved in synthesis	Fungal filtrate mixed with AgNO_3_ at 28 °C for 28 days then treated with KOH or NaOH for 5 days	[191]
	*Aspergillus niger* *Trichoderma longibrachiatum*	Spherical, cylindrical, and oval shapes 18–77 nm for *A. niger* AgNPs 15–54 nm for *T. longibrachiatum*	Antimicrobial, antioxidant, catalytic, anticoagulant, Thrombolytic agents Dye degradation Potiential use of fungal xylanase for AgNPs synthesis	xylanases synthesized by fungal strains mixed with AgNO_3_ at 30 °C for 5 min.	[192]
	*Marine fungus Cladosporium halotolerans*	Spherical AgNPs	Antioxidant Antifungal Dose dependent cytotoxic activity		[193]
	*Pleurotus djamor var. roseus*	Spherical shaped 5–50 nm	Anti-proliferative agent against prostate carcinoma cells	Filtered fungal extract was mixed with AgNO_3_ for 12 h.	[194]
	*Inonotus obliquus*	Spherical 14.7–35.2 nm	Antibacterial and anti-proliferative in cancer cells	Mushroom extract mixed with AgNO_3_ for 8 h.	[195]
AuNPs	Pathogenic *Chrysosporium tropicum*	Spherical shaped 20–50 nm	Larvicide against the *Aedes aegypti* larva	Filtered fungal extract mixed with HAuCl_4_ at 25 °C for 72 h.	[196]
	Yeast cells of *Magnusiomyces ingens* LH-F1	Sphere, triangle and hexagon 16–423 nm	Efficient catalysts for the reduction of nitrophenols.	Filtered fungal biomass mixed with HAuCl_4_ at 30°C for 24 h.	[197]
	*Aspergillus sydowii*	Spherical 8–15 nm	–	filtered fungal extract mixed with AuCl_3_ at 27 °C for 72 h.	[198]
	Cell surface proteins of *Rhizopus oryzae*	Spherical 16–19 nm	No harsh chemicals or multistep preparation required for synthesis or separation favoring environmental sustainability Good hemocompatibility and colloidal stability	Extraction of cell surface proteins from *R. oryzae* using sodium dodecyl sulfate (SDS) Triton X-100, and 1,4-dithiothreitol prior to mixing with Au precursor	[199]
TiO_2_-NPs	*Aspergillus flavus*	Spherical Oval Aggregated 62–74 nm	Antimicrobial activity against *E. coli*	Mycelial filtrate mixed with TiO_2_ at 37 °C	[200]
	*Baker's yeast.*	Spherical 6.2 nm	Antibacterial against *S. aureus* Antifungal against *C. albicans*	TiCl_3_ solution added to 24 h old yeast culture, while stirring until a clear purple solution	[201]
	*Aspergillus tubingensis* TFR-5	Downy outer surface and showing monodispersity 1.5–30 nm	Plant nutrient fertilizer to enhance crop production	Mycelial filtrate mixed with TiO_2_ for 28 °C for 36 h.	[202]
IONP	*Saccharomyces Cerevisiae (baker's yeast)*	Spherical 155.7 nm	Antibacterial activity against Gram-positive *B. sutblis*	Ferric chloride/Ferrous sulphate solution mixed with freshly prepared cultures of yeast at 30°C for 2–3 days	[203]
	*Penicillium roqueforti MK805460.1*	Spherical 5–15 nm	Antibacterial activity against	–	[204]
CuO and ZnO-NPs	*Aspergillus terreus* strain AF-1	Spherical 11–47 nm	Antibacterial activity (used with cotton fabrics)	Fungal biomass filtrate mixed with CuSO_4_·5H_2_O for 24 h at 28 °C	[186]
	*Trichoderma harzianum*	CuO: Dispersed and elongated fibres 38–77 nm ZnO: fan and bouquet structure 27–40 nm	Antifungal activity against *A. alternata* and *P. oryzae*	Cell free mycelia filtrate were mixed with CuSO_4_·5H_2_O and ZnSO_4_·7H_2_O at 45 °C for 6–12 h.	[187]

## Fungal-derived antimicrobial metal nanoparticles

The biggest problem with external fracture fixation is pin-tract infections (PTIs) where the inserted pin allows bacterial and biofilm adherence [205]. Most treatments have proven ineffective and pin removal is necessary. In an effort to overcome PTIs, recent developments suggest the usage of anti-infective coatings with antibacterial activity [206]. Liang *et al.* facilitated an ecofriendly large-scale production of SeNPs using *Aureobasidium pullulans*. Following cultivation, *Aureobasidium pullulans* supernatant was mixed with sodium selenite under shaking condition at 25 °C for 72 h. prior to SeNPs harvest. Granular SeNPs (50–70 nm) were obtained and doped on silver coating to create silver-selenium (Ag-Se) coatings. The bactericidal activity of Ag-Se-NPs coatings on *S. aureus* and *E. coli* were comparable to commercial AgNPs and Ag-PTFE-coated surfaces. They were also corrosion resistant [207].

The mycelia extract of *Aspergillus melleus* can fabricate biogenic AgNPs with eminent anticancer and antibacterial vigor. On MG-63 cells, *A. melleus* AgNPs have significant cytotoxic capability. *S. aureus* and *E. coli* were also sensitive to *A. melleus* AgNPs' [208]. The mycelia extract of a soil derived *Aspergillus niger* can synthesize ZnO-NP with promising anticancer and antibacterial activity. The purified ZnO-NPs are rod shaped (11–17 nm) with bactericidal activity against *S. aureus, Salmonella enterica, Bacillus cereus*, and *E. coli*, *Aspergillus niger*. Additionally, Vancomycin and *A. niger* ZnO-NPs demonstrated strong synergistic antibacterial vigor against tested organisms. They also acquire potent anticancer activity against A549 cell line with an IC_50_ score of 50 μg/ml [209]. *A. niger* ZnO-NPs also exerts strong antioxidant and cytotoxicitc effects on human hepatocellular carcinoma cells (HepG2) [210].

Due to their distinctive physico-chemical (catalytic, magnetic, and optical) and biological (antimicrobial, antioxidant and anticancer) capabilities, platinum nanoparticles (PtNPs) are of tremendous interest in a variety of technical and biomedical sectors. Bio-fabrication of PtNPs by *F. oxysporum* is studied by Riddin *et al.* to identify optimal condition for biosynthesis. Intracellular, extracellularly as well as hyphael surface synthesizes generated different shapes of PtNPs (hexagons, pentagons, circles, squares and rectangles with average sizes 10–100 nm). However, only the extracellular production was found to be statistically significant [211]. Using *F. oxysporum* filtrate, Gupta and Chundawat produced face-centered cubic PtNPs (25 nm) with antibacterial and photocatalytic activities [212]. Highly diffusible non-aggregating PtNPs (5–40 nm) are also achievable from *Penicillium chrysogenum* culture filtrate [213]. *Neurospora crassa* is another ascomycota that can synthesis platinum nanoparticles with different sizes and shapes. Extracellular PtNPs (4–35 nm) and spherical nanoaggregates (20–110 nm) are formed when mycelial biomass was incubated with H_2_PtCl_6_. Further, round single crystal nanoagglomerates (17 to 76 nm) and individual single crystals (2–3 nm) can also be produced using mycelial extract of *Neurospora crassa* [214]. Further, the phytopathogenic fungus *Alternaria alternata's* culture filtrate has been used to produce nanoplatinum of quasi-spherical, rectangular, tetrahedral, hexagonal and polygonal morphologies (∼135 nm) [215].

When combined with ciprofloxacin, *Candida albicans* silver nanoparticles produced by Bhat *et al.* exerted effective antibacterial vigor against various bacterial pathogens. *C. albicans* AgNPs are bactericidal and synergistic effects were observed when combined with ciprofloxacin [216]. Butyl acrylate caped AgNPs purified from culture extracts of *Alternaria alternate* were doped in cotton to generate nanoparticle treated cotton that can combat *E. coli* and *S. aureus*. The cottons impregnated with biogenic AgNPs inhibit bacterial growth at different concentrations [217]. The oropharyngeal *Candida glabrata* isolates synthesize AgNPs with exquisite bactericidal and fungicidal activity [218].

Biogenic AgNPs have also demonstrated antiviral properties. Using *Alternaria* sp., *Fusarium oxysporum, Curvularia* sp., *Chaetomium indicum*, and *Phoma* sp., Gaikwad *et al.* created silver nanoparticles that inhibited the reproduction of HSV-1, HSV-2 and HPIV-3 in cell cultures. The most efficient and least harmful nanoparticles were generated by *F. oxysporum, Curvularia* sp. and *C. indicum* [219]. When tested on the larvae and pupae of the dengue vector mosquito *Aedes aegypti*, Sundaravadivelan and Padmanabhan biogenic AgNPs purified from the filtrate of *Trichoderma harzianum* exerted dose dependent larvicidal effects [220]. Another study by Banu and Balasubramanian examined the effectiveness of AgNPs retrieved from the entomopathogenic fungus *Isaria fumosorosea* in controlling the mosquito species *Culex quinquefasciatus* and *A. aegypti*. *Isaria fumosorosea* AgNPs demonstrated larvicidal effects [221].

## Fungal-derived anticancer metal nanoparticles

Intracellular fabrication of AgNPs facilitated by co-cultivation of *A. niger* biomass with AgNO_3_ yields capped spherical nanoparticles with anticancer activities. These AgNPs suppressed 70.2% of the HeLa cell growth [222]. Nassar *et al.* bio-fabricated SeNPs from the culture filtrates of endophytic *Penicillium verhagenii* co-incubated with metal salt precursor (Na_2_SO_3_) at 25 °C overnight. The purified SeNPs were spherical and crystalline in nature. Interestingly, the biogenic *P. verhagenii* SeNPs elucidated antimicrobial vigor against various Gram negative and Gram-positive strains with MIC scores ranging from 12.5–100 μg/ml. It was also noted that *P. verhagenii* SeNPs acquired a dose dependent antioxidant DPPH-scavenging capability. Moreover, the *P. verhagenii* SeNPs were lethal to *in vitro* cultured PC3 and MCF7 cancer cell lines with IC_50_ values of 225 and 283 μg/ml, respectively, and biocompatible with normal WI38 and Vero cell lines. They were also larvicidal to *Aedes albopictus* instar larvae [223].

Fouda *et al.* utilized the endophytic fungal species *Penicillium crustosum* to achieve spherically crystalline SeNPs. These SeNPs elucidated antimicrobial activities while experimenting with different bacterial strains that were enhanced with light irradiation. It also acquired anticancer activity when incubated with *in vitro* cultured T47D and HepG2 cell lines. Their anticancer activity was also enhanced following exposure to light where the cell viability % scored in dark conditions was reduced from 95 and 93% to 84 and 46%, respectively. Impact of light irradiation was also noted when the IC_50_ scores were lowered from 109 to 70 μg/ml for the T47D and 19 to 4.8 μg/ml for the HepG2 post light irradiation. Irradiation also elevated SeNPs photoluminescence activity adding a dye degrading ability to its activity profile. The *Penicillium crustosum* SeNPs elucidated exceptional durability and reusability [224]. The filtered biomass of marine Fungi, *Hamigera Pallida*, is also utilized to synthesize biogenic AgNPs from AgNO_3_. The resultant AgNPs were spherical (3–16 nm) with significant antibacterial vigor against different bacterial strains and potent antioxidant capabilities. With an IC_50_ value of 66 μg/ml, the AgNPs are cytotoxic to human breast cancer (MCF-7) cell line [225].

The antibacterial and anticancer potential of AgNPs produced by *Fusarium oxysporum* was also assessed by Husseiny *et al.* The nanoparticles suppressed tumor cell line (MCF-7), *E. coli*, and *S. aureus*. According to Husseiny *et al.*, the effect was attributed to the silver nanoparticles' involvement in the disruption of the mitochondrial respiratory chain, which produced reactive oxygen species and prevented the synthesis of ATP [226].

## Green metal nanoparticles produced by viruses

Virus can be used in bio-reduction of metal ions to MNPs and can create 3D nanomaterials for targeted medication delivery [227]. Viral modification allows the deposition of materials inside the capsid core of viruses. Viruses can also produce nano-conjugates and nano-composites. For instance, AuNPs and AgNPs (32 nm) are fabricated by the phytopathogenic virus Squash leaf curl China virus (SLCCNV). These eco-friendly SLCCNV-metallic-hybrid nanoparticles are produced within 5 min period only. The nanoparticles elucidated remarkable electrical conductivity which made them eligible for several biomedical applications [228].

Small amounts of tobacco mosaic virus (TMV) and bovine papillomavirus (BPV) were added to different kinds of plant extracts as additives to enhance the production of nanoparticles. A notable rise in the metal ion reduction capacity of aqueous plant extract was observed post addition of these viruses [24]. Genetically modified TMV, MBP-TMV, can produce distinct gold nanoparticles (10–40 nm) when mixed with 3 mM potassium tetrachloroaurate. The MBA-TMV-AuNPs elucidated high stability and crystalline structure [229]. To construct gold nanoparticles with interparticle spacing, several variants of the Cowpea Mosaic Virus (CPMV) have been employed as scaffolds for the attachment of 2 and 5 nm AuNPs through the creation of gold–sulfur bonds at certain sites on the virus. The generated AuNPs suggest that CPMV mutants can serve as nanoscale scaffolds [230]. In chemotherapy and photothermal therapy, viruses were utilized to transport medications and control release. Red Clover Necrotic Mosaic Virus (RCNMV) was used by Cao *et al.* to create nanoparticles that release doxorubicin during chemotherapy [231].

## Green metal nanoparticles produced by algal species & their biological applications

Phycosynthesis of MNPs is mediated by algal species. Algae are polyphyletic, autotrophic and photosynthetic eukaryotic creatures found in marine, and freshwater environments and on damp rocks. They are classed according to their morphological traits into microalgae and macroalgae. MNPs phycosynthesis is facilitated by the high abundance of polyunsaturated fatty acids, chlorophyll, carotenoids, tocopherols, polyphenols and phycobilins that act as stabilizing/capping and reducing agents for nanoparticles [232]. Extracellular MNPs phycosynthesis is initiated by mixing metal ion precursor solution with the boiled algal extract until the color changes [233]. It's the best method for the generation of MNPs by algal biomasses. Intracellular MNP production by algae involves the usage of the live algal biomass itself (after collection and washing) followed by sonication to harvest the intracellular MNPs [234]. Here, the NADPH dependent reductase serves as reducing agent. Intracellular synthesis of AuNPs is initiated by the co-cultivation of *Ulva intestinalis* and *Rhizoclonium fontinale* with chloroauric acid at 20 °C for 72 h until the green color turned purple [232,235]. Color changes observed in *Klebsormidium flaccidum* in a silica gel suspension denotes the reduction of gold precursor which is confirmed by presence of dark-colored patches in thylakoid membrane TEM examination [232,233,235].

Recently, ultrasonic irradiation-assisted synthesis (UIAS) of MNPs by *Cyanobacterium* sp. elucidated accelerated chemical reactions rates and eased MNPs extraction processes. This is attributed to the cavitation collapses induced by ultrasonic waves causing severe local heating (5000 °C) and high pressures (2000 °A) within the liquid reaction mixture that markedly shortened MNP synthesis reaction time [236]. *Euglenoids* and diatoms, as well as other types of algae like *Phaeophyceae, Cyanophyceae* and *Rhodophyceae*, have been used in the fabrication of metallic NPs including palladium, gold, iron and silver [233]. The brown algae species *Turbinaria conoides* and *Padina tetrastromaticaare* seaweed are AgNPs producers. The produced AgNPs are spherical in shape (96 nm) and bactericidal [237]. The spherical poly-dispersed AuNPs are also fabricated by *Sargassum myriocystum, Sargassum wightii, Cystoseira baccata, Stereospermum marginatum, Ecklonia cava* and *Padina gymnospora* [238].

The *Lemanea fluviatilis*, marine red algae, are potential producers of AuNPs using chloroauric acid [233]. Red algae *Gracilaria edulis* are capable of producing bimetallic Ag–Au nanoparticles with powerful anti-cancer activity against human breast cancer cells utilizing various molar ratios of HAuCl_4_ and AgNO_3_ [239]. The blue algae, *Spirulina platensis*, secrete both AgNPs and Au-NPs of spherical, octahedral and cubic shapes utilizing different proteins and peptides as reducing agents [240]. *Chlamydomonas reinhardtii* promotes the synthesis of cadmium sulphide bimetallic nanoparticles (CdSNPs), which have numerous uses in fields including biosensors, LEDs, and photo-catalysis [241]. The green macroalgae are capable of producing metallic nanoparticles. One of the most advantageous types of green macroalgae, *Ulva fasciata*, was used to produce ZnO-NPs with antibacterial qualities [242]. In a different investigation, octahedral ZnO-NPs and spherical AgNPs were produced using *Gracilaria edulis* [243].

### Algal-derived antimicrobial metal nanoparticles

*Padina tetrastromatica* AgNPs are bactericidal to *B. subtilis, K. planticola* and *P. aeruginosa* [244]. *Caulerpa serrulata* AgNPs acquire stable and colloidal morphologies with remarkable antimicrobial activity [245]. In similar vein, AgNPs retrieved from aqueous extract of *Pithophora oedogonia* are bactericidal to a number of bacterial pathogens, including *B. subtilis, Micrococcus luteus, V. cholerae, S. aureus* and *E. coli* [246]. The *Stoechospermum marginatum* derived AuNPs elucidate superior antibacterial activity against *E. faecalis* when compared with the standard tetracycline antibiotic [247]. The *Dictyosphaerium* sp. and *Pectinodesmus* sp. AgNPs are also anti-bacterial to 14 bacterial species, antifungal to *C. albicans*, and cytotoxic toward hepatocellular carcinoma (HepG2), breast cancer (MCF7) and Newcastle disease virus (NDV) in Huh7-infected cells [248]. Additionally, Venkatesan *et al.* showed that the brown seaweed *Ecklonia cava* extracts utilized for biogenic fabrication of AgNPs have significant anti-bacterial activity against *Escherichia coli* and *Staphylococcus aureus* and antioxidant apoptotic activity toward *in vitro* cultured human cervical carcinoma (HeLa) cells [249].

## Factor affecting green synthesis of metal nanoparticles

Adjustment of several physical conditions including metal precursor concentration, pH, temperature, culture biomass and reaction time is crucial to optimize the production of MNPs through biological processes. Variation in the pH during phyto-synthesis of MNPs is associated with variation in MNPs particle size. Acidic pH is associated with higher yield of large sized MNPs while smaller MNPs particle sizes are observed in alkaline medium pH [250]. Biogenic AgNPs tend to be spherically shaped; however, at pH 5 and 11, biogenic AgNPs tend to acquire triangular and hexagonal forms. Further, AgNPs retrieved from alkaline and neutral medium tends to be more stable as raising the pH increases the number of negative charges on their external surfaces. Negatively charged AgNPs have reduced tendencies to aggregation due to electrostatic repulsion [251]. Altering pH (2 to 10) during the AgNPs synthesis by brown algae, *Sargassum angustifolium*, was associated with stable uniformly sized AgNPs at alkaline pHs (pH 10) [252].

Different reaction times are associated with differences in MNPs yield and particle size. The extracts of *Ananas comosus* generate MNPs within 2 min but optimal spherical MNPs (12 nm) are observed within 5 min of bioreduction [253]. Furthermore, Aboelfetoh *et al.* (2017) demonstrated that the increasing the contact time between *Caulerpa serrulata* and silver ion (Ag^+^) at room temperature results in rapid synthesis of non-agglomerated AgNPs [235]. The growth kinetics of AgNPs generated by aqueous extract of *Azadirachta indica* (Neem tree) leaves were studied by Prathna *et al.* [254]. The reaction was carried out for 4 h while being examined using UV-Vis (ultraviolet-visible) spectrometry. Within 2 h, the first peak of the UV-Vis spectrum became visible, indicating synthesis of MNPs within the size range 10 to 35 nm. Neem AgNPs were spherically shaped (∼20 nm) after 2 h. The size of Neem AgNPs rose to 36.6 nm when the reaction time reached 4 h [254]. Further, the reaction time affects the aggregation rate and magnetic properties of MNPs. It was reported by Karade *et al.* that increasing the reaction time was associated with larger MNPs and enhancement in saturation magnetization [255].

Incubation temperature also affects synthesis rate and morphologies of biogenic MNPs. Higher MNP production rates were observed upon increasing temperatures [256]. Fast reduction of biogenic AgNPs by *Vitex agnus-castus* leaves extract took place at 40 °C while higher AgNPs yield occurred at higher temperatures (60–80°C) [257]. Varying the concentration of metal ion precursor and biological biomasses also affect the overall performance and yield of MNPs. The concentration of metal ion salt precursor plays a significant role in determining the size of biogenic MNPs, since higher concentrations are associated with MNPs agglomerations and aggregation [258]. Bio-fabrication of MNPs is also highly influenced by concentration of reducing agents in the biological biomasses. For instance, production of AuNPs by the *Aloe vera* leaf extracts was highly dependent on the concentration of multiple twinned particles (MTPs) [259]. Furthermore, leveling up extract concentrations of marine green macroalga *Caulerpa serrulata* to a solution of constant concentration of silver nitrate at room temperature lowered the average size of AgNPs [235].

The choice of the organism and its storage condition are also crucial to consider during green biogenic synthesis of MNPs. It's crucial to determine the right inoculum size and whether adding a biocatalyst to the culture filtrates can help with the bio-reduction of metal ions. Additionally, using complete microbial or plant cells is preferred since they have their own enzymes or co-factors (such NADH, NADPH or FAD) that can operate as biocatalysts [234].

## Challenges & optimizations of green metal nanoparticles

There are some obstacles that stand in the way of industrial and commercial application of green biological synthesis of MNPs. First, maintenance of the size, shape regularity and dispersion of green synthesized MNPs is essential and requires optimization. It is well established that the behavior and properties of MNPs are significantly influenced by the particle size and dispersity (size distribution). A monodispersed population of MNPs is required for commercial applications as opposed to the poly-dispersed MNPs [260]. It's also known that dispersion level and size of MNPs affect the overall biomedical activity. For instance, anticancer efficacy of 50 nm silica NPs-drug conjugate was higher than that of 20 and 200 nm [261]. Smaller-sized AgNPs were more successful at inducing apoptosis than larger-sized AgNPs [262]. Mono-dispersed MNPs can be produced using a variety of optimization strategies, including adjusting the pH, temperature, precursor salt content and culture growth conditions [24]. Table 5 summarizes some of the optimization strategies applied to maintain regularity of green synthesized nanoparticles.

**Table 5.** Examples of optimization strategies implemented to enhance characteristics of green metal nanoparticles.

Organism utilized for green synthesis	Utilized species	MNP	Factors optimized	Favorable outcomes	Advantages of biogenic MNP	Ref.
Bacteria	Soil bacteria *Leclercia adecarboxylata THHM*	AgNPs	• pH • Conc. of silver salt precursor solution (AgNO_3_) • Incubation time • Incubation temp. • Illumination condition • Conc. of bacterial filtrate	Incubation time of 72.0 h with 1.5 mM silver nitrate at 40.0 °C, pH 7.0, and a supernatant conc. 30% (v/v) under illumination	Monodispersed with ∼17 nm size Antibacterial and antifungal activity	[263]
	*B. subtilis NRC1*	AuNPs	Influence of nitrogen, carbon sources and metal ions were screened using one variable at a time (OVAT) method	Optimum conditions to increase AuNPs production by 2.6-fold: 0.74% (w/v) casein hydrolysate, 3.99% (w/v) dextrin, 47 × 10^6^ CFU/ml, pH 7.76, at 25°C	Increased AuNPs yield with confidence factor 98.48%	[264]
	*Halomonas elongata*	SeNPs	For max. activity of SeNPs on *Candida albicans* growth, these factors were tested: • Sodium selenite concentration • Glucose concentration • Incubation time	70% inhibition of Candida albicans growth was observed with: 0.8 mg/ml Na selenite, 7.5 mg/ml glucose at 48 h. incubation	Effective antifungal and antibacterial agent for mouthwashes	[265]
Fungi	*Penicillium* sp.	AgNPs	Optimization of fungal growth conditions for optimal production of AgNPs (pH, biomass size, conc. of precursor salt, Temp., and incubation period)	For enhanced AgNPs production: pH 8, 37 °C, at 2 mM AgNO_3_ and 20 g of wet biomass.	Spherical well-dispersed 4–55 nm Antibacterial Cytotoxic agent	[266]
	*Agaricus bisporus*	AuNPs	Optimization of AuNPs production included: • Conc. of AuCl_3_ • Ratio of AuCl_3_ to volume of a mushroom extract, • pH • Temperature • Reaction time	Optimization conditions for max. production of AuNPs: pH 7, 1 mM HAuCl_4_, ratio of (1:9) mushroom extract to HAuCl_4_, incubate for 72 h at dark and 28°C	Spherical Monodispersed 10–50 nm Antibacterial and antifungal	[267]
Plant	*Eucalyptus camaldulensis* *Terminalia arjuna* extracts	AgNPs	Optimization of AgNPs production: • Conc. of AgNO_3_ • Incubation time • Incubation Temp. • pH	Max, amount of AgNPs using: 1 mM AgNO_3_ at 75 °C for 60 min.	Spherical Monodispersed Antibacterial activity	[268]
	*Salvia africana-lutea Sutherlandia frutescens*	AuNPs	Optimization of AuNPs production: • Incubation time • Temperature • Plant extract conc. • AgNO_3_ conc. • Sodium tetrachloroaurate (III) dihydrate (NaAuCl_4_ · 2H_2_O) conc.		Antibacterial activity	[269]

## Future perspective in the green synthesis of metal nanoparticles

Terpenoids, alkaloids, polyketides and flavones are abundant in marine and soil actinomycetes. Marine actinomycetes secondary metabolites share chemical similarities with a number of phytochemicals that various algal and plant species used during the green MNPs synthesis [270]. Actinomycetes are the least studied green nano-factories. Therefore, marine actinomycetes may be introduced as new generation in green MNPs nanofactories [271]. Khalil *et al.* utilized the silver tolerant *Nocardiopsis dasonvillei* KY772427, for the fabrication of biogenic AgNPs. Interestingly, *Nocardia sp.* AgNPs are mono-dispersed, spherical and acquired negative surface charge. They exhibited synergistic effects with antimicrobial agents (peracillin-tazobactam and fluconazole) against pathogenic Gram-positive (*S. aureus*), Gram-negative bacteria (*P. aeruginosa*), and fungal strains (*A. niger, C. albicans*). The *Nocardia* AgNPs also acquired potent insecticidal activities against *Macrosiphum rosae*, antioxidant and anticancer activities against CaCO-2 cells [272]. The culture filtrates of actinomycete strains retrieved from sea sponge *Crella cyathophora* successfully generated AgNPs with significant antibacterial activity against *P. aeruginosa* and *E. cloacae* [273].

In a similar vein, Abd El-Ghany *et al.* isolated biogenic AgNPs from rare silver resistant actinomycete strains, *Glutamicibacter nicotianae* SNPRA1 and *Leucobacter aridicollis* SNPRA2. The SNPRA1 and SNPRA2 AgNPs were fungicidal to *A. flavus* and *Aspergillus ochraceus* with acceptable biocompatibility on human skin fibroblast cells suggesting their safety and tolerability [274]. The *Streptomyces* sp. SSUT88A AgNPs elucidate potent antibacterial vigor that outperformed commercially available AgNPs against multidrug resistant *Acinetobacter baumannii*. It was also effective against other Gram-negative species, including the *E. coli, K. pneumoniae* and *P. aeruginosa* [275]. Zhao *et al.* utilized the culture supernatants of marine endophytic actinomycetes CKV1 as reducing and stabilizing agents for copper sulfate (CuSO_4_.5H_2_0) to generate green CuO-NPs. The CKV1 CuO-NPs were spherical (∼20 nm) and elucidated eminent antibacterial vigor against biofilm producing Gram-negative *E. coli* and *P. mirabilis*. The CKV1 CuO-NPs are also lethal to *in vitro* cultured A549 lung carcinoma cell lines [276].

Statistical optimizations utilizing response surface methodology (RSM) and multifactorial design are supportive methods that have been newly undertaken for the production optimization of various secondary antibacterial metabolites [277,278], antifungal metabolites [279] and biosurfactants [280,281]. Therefore, this approach is promising during the optimization procedures of green synthesized nanoparticles.

Introduction of whole genome analyses during green MNP synthesis is essential. The metabolic characteristics of synthesizing strains will be better understood with the aid of thorough genomic investigations, which will also assist the identification of enzymes/metabolites involved in metal ion reduction and ease the scaling up of green MNP synthesis [282]. Using a bacterial cell-free extract (CFE) facilitate easy purification of MNPs, much easier to standardize, optimize and replicate during large scale productions. The CFEs are also opulent with proteins, amino acids, peptides and enzymes that can act as the phytochemicals, serving as capping, reducing and stabilizing agents [283].

## Future perspective for green metal nanoparticles in biological therapies

Biogenic SeNP nanoparticles that showed enhanced activities post exposure to light irradiations can be successfully coupled with antimicrobial photodynamic therapy (ADPT). The ADPT is a promising non-antibiotic based method that's utilized to combat MDR infectious diseases. Briefly, ADPT depends on a photosensitizer (PS), a nontoxic dye, along with a safe visible light. When the PS is irradiated, ROS are released and several cellular components are irreversibly damaged. Due to the multi-target nature of ROS species, the chances of developing microbial resistance against ADPT are almost absent [284]. Previous reports denoted the effectiveness of combining several MNPs with ADPT. TiO_2_ nanoparticles are considered photocatalytic antimicrobial agents and have been used in various coatings. Upon irradiating TiO_2_ coated surfaces with UV rays at 385 nm, free radicals are released initiating bacterial lysis. UV irradiated TiO_2_-NPs solutions might be an effective strategy to get rid of viruses including hepatitis B, influenza subtypes, poliovirus and coronavirus. Fungi such as *A. niger, C. albicans and Cryptosporidium parvum* can be effectively eliminated by UV irradiated TiO_2_ [285]. Since Fouda *et al.* had previously reported that UV irradiation of biogenic SeNP enhanced both its cytotoxic and antibacterial activities, conjugating these nanoparticles with ADPT would be beneficial to combat MDR bacteria [224].

For their intriguing physiochemical and optical characteristics, green MNPs (Fe, Co, Ni, Ag, Au, Mn) can replace chemically synthesized MNPs in cancer combination therapies in particular hyperthermia and radiotherapy. The most important variables that affect hyperthermic intensity of MNPs are particle size, NP dispersity and magnetic moment [286]. It has been previously reported that gold nanoshells improved the efficacy of thermal therapy in combination with radiotherapy for their unique optical, electrical, and drug loading capacities [287]. The cobalt ferrites (CoFe_2_O_4_), manganese ferrite (MnFe_2_O_4_), nickel ferrite (NiFe_2_O_4_), and lithium ferrite (Li0.5Fe_2_.5O_4_), provide the foundation for another set of MNPs used in hyperthermia treatment for their quite high heating capability (1300–1600 W/g) [288].

Additionally, the toxicities of green MNPs should be taken into consideration. In order to fully comprehend their mode of action and associated side effects, more *in vivo* studies must be carried out. Kobylinska *et al.* green FeCl_3_/FeSO_4_/CoCl_2_ mixture purified from the haiy root extract of *Artemisia tilesii* exhibited supermagnetic properties with no phyto-toxicities against *Cichorium intybus and Lactuca sativa* plants which are known for their distinguishably high and active seed germination rates. The seeds are thought to be more sensitive to metal toxicities and the study denotes that green oxide MNPs have no effect of germination rates of seeds suggesting their environmental safety [289].

## Conclusion

Incorporating MNPs into various biological processes has become standard practice that aim to improve therapeutic outcomes and overcome some of the drawbacks associated with many conventional therapies. To overcome the drawbacks of conventional chemical and physical MNPs synthesis, a new area of nanotechnology utilizes green synthesis to create MNPs have emerged. Since it avoids various hazardous elements associated with both chemically and physically synthesizing procedures, green synthesis is extremely advantageous for nanomedicine. It is efficient, eco-friendly and simple to execute. By utilizing a variety of species and carrying out the synthesis under varying temperature, pH, biomass, and metal precursor concentration conditions, it is feasible to produce MNPs with favorable physicochemical and biological features. Numerous biogenic MNPs have shown a variety of biological activities in diverse *in vitro* investigations, nevertheless these MNPs face a number of challenges when it comes to maximizing their synthesis and figuring out their mode of action and associated side effects. Overall, green manufacturing of metal/metal oxide nanoparticles supports a variety of biological therapies, including the control of antioxidant, antifungal and antibacterial activities.
